# Artificial Intelligence: Impact and Challenges to Authors, Journals and Medical Publishing

**DOI:** 10.5704/MOJ.2311.001

**Published:** 2023-11

**Authors:** WCG Peh, A Saw

**Affiliations:** 1 Department of Diagnostic Radiology, Khoo Teck Puat Hospital, Singapore; 2 Department of Orthopaedic Surgery (NOCERAL), University of Malaya, Kuala Lumpur, Malaysia

## Abstract

Artificial intelligence (AI)-assisted technologies are here to stay and cannot be ignored. These tools are able to generate highly-realistic human-like text and perform a wide range of useful language tasks with a wide range of applications. They have the potential to expedite innovation in health care and can aid in promoting equity and diversity in research by overcoming language barriers. When using these AI tools, authors must take responsibility for the output and originality of their work, as publishers expect all content to be generated by human authors unless there is a declaration to the contrary. Authors must disclose how AI tools have been used, and ensure appropriate attribution of all the text, images, and audio-visual material. The responsible use of AI language models and transparent reporting of how these tools were used in the creation of information and publication are vital to promote and protect the credibility and integrity of medical research, and trust in medical knowledge. Educating postgraduate and undergraduate students, researchers and authors on the applications and best usage of AI-assisted technologies, together with the importance of critical thinking, integrity and strict adherence to ethical principles, are key steps that need to be undertaken.

## Editorial

Artificial intelligence (AI) is a field which aims at problem-solving by combining computer science with robust datasets. Machine learning, a branch of AI, uses data and algorithms to imitate the way humans learn, gradually improving its accuracy with time^1^. Natural language processing (NLP) is another branch of AI that gives computers the ability to understand text and spoken words, similar to humans. These technologies enable computers to process human language in text or voice data form and to understand its full meaning, together with the writer’s or speaker’s intent or sentiment^2^. A large language model (LLM) is an AI algorithm that uses deep learning techniques and massive data sets to understand, summarise, generate and predict new content. LLM can be considered a type of generative AI that specifically generates text-based content^3^. A chatbot is a computer program that uses AI and NLP to understand questions or prompts, and automate responses to them, simulating human conversation. Chatbots continue to evolve and improve, and by using advanced AI tools, are currently able to discern the user’s needs to determine what the user is trying to accomplish. Based on machine and deep learning, as well as continued human user interactions, chatbots have increasing ability to predict user’s needs more accurately and respond more correctly over time^4^.

In November 2022, OpenAI, an American company based in San Francisco, released a new open-source NLP tool called ChatGPT. GPT stands for “generative pretrained transformer”. ChatGPT is an evolution of an earlier chatbot (“InstructGPT”) that was trained to follow an instruction in a prompt or question and then provide a detailed response. ChatGPT interacts in a conversational way, with its dialogue format making it possible for ChatGPT “to answer follow-up questions, admit its mistakes, challenge incorrect premises, and reject inappropriate requests”^5^. As Open AI made ChatGPT user-friendly, easily accessible and free-to-use, ChatGPT became an instant hit, with millions of people worldwide trying out and using this new tool. Following ChatGPT’s success, other companies have also developed conversational AI models^6^. Since its inception, ChatGPT has dramatically impacted general and healthcare education, academic and scientific writing, research and medical practice. ChatGPT has shown numerous benefits, including the ability to write presentable student essays, summarise research papers, do literature reviews, generate codes and answer questions well enough to pass medical exams. It can help streamline clinical workflow, documentation, and improve health literacy by providing easily accessible and understandable health information to the general public. It has even produced good enough research abstracts that scientists find difficult to determine whether or not they have been generated by a computer or human^7-10^. LLM may be able to remove language barriers by enabling more people from non-English speaking nations to write high-quality text^6^.

However, the use of ChatGPT and other AI-assisted technologies has raised concerns about its limitations and dangers, particularly issues relating to the authenticity and credibility of academic work. Authors of scientific papers have certain responsibilities that cannot be assumed by any computer or AI programme6. Since the recent rise of ChatGPT, an increasing number of journals, publishers and leading bodies such as the International Committee of Medical Journal Editors (ICMJE) and World Association of Medical Journal Editors (WAME) have produced guidelines advocating ethical use of AI-assisted technologies, such as chatbots, LLMs or image creators^9,11-14^. Chatbots and other AI-assisted technologies cannot be listed as an author of a paper because being non-human (hence, not a legal person), they do not fulfil the ICMJE authorship criteria. They will not be able to undertake responsibilities required for authorship, i.e. vouching for accuracy, integrity and originality of the work; “giving final approval of the version to be published” and “to be accountable for all aspects of the work in ensuring that questions related to the accuracy or integrity of any part of the work are appropriately investigated and resolved”^13,14^. Similarly, AI tools will not be able to understand or legally sign a conflict-of-interest statement14. Several preprints and published articles have already credited ChatGPT with formal authorship!^9^. In January 2023, the Elsevier journal Nurse Education in Practice sparked a huge debate when it published an article listing ChatGPT as a co-author, but subsequently published a corrigendum removing ChatGPT and leaving a human as the sole author^15,16^.

Authors need to carefully review and edit all material because although AI can generate authoritative-sounding output that are often linguistically accurate and convincingly fluent, this output can be incorrect, incomplete or biased^13,14^. Scientific essays that appear credible may contain a mix of true and completely fabricated generated data^17^. Chatbots and other AI-assisted technologies are highly dependent on algorithms and the contents of materials used in its training, and hence retain the information supplied to them for possible use in future responses. Problems such as unintentional plagiarism and misattribution of concepts are real concerns, and irrelevant content may be incorporated into the generated output^14^. As a chatbot may be designed to omit sources that oppose viewpoints expressed in its output, bias may be introduced in its output. It is therefore the authors’ responsibility to seek out, review and include such counterviews in their articles^14^. One current limitation of ChatGPT is that it is only able to generate text based on the input provided to it, i.e. it does not have access to external information or the ability to browse the internet. This means that ChatGPT will be unable to provide accurate or up-to-date information on a wide range of topics^18^. ChatGPT may also not be able to generate responses to complex or unconventional questions.

Artificial hallucination is a phenomenon where a chatbot produces output that is not real. These occur when generative AI models have been trained on large amounts of unsupervised data that contain biases and misinformation. These biases are incorporated and amplified, resulting in artificial hallucinations that perpetuate false information^17^. Some AI tools also fabricate references, something that reviewers and editors need to be cognisant of during the review process^19^. ChatGPT can provide references to specific questions, where the titles are non-existent, and the provided PubMed ID (PMIDs) are of unrelated papers^17^. The ability of chatbots to retain and reuse supplied information also impacts on confidentiality of accessed documents, a pitfall that authors, reviewers and editors should bear in mind when using such AI technologies^14^.

Humans are expected to be accountable for any submitted material that includes the use of AI-assisted technologies^13^. At time of manuscript submission, authors should transparently disclose whether they have used AI-assisted technologies in the production of their submitted work and how these have been used. It is recommended that details such as all prompts used to generate new text, or to convert text or text prompts into tables or illustrations, be specified. Use of any AI tool (including the name of the model or tool, version and extension number, and manufacturer) to carry out or generate analytical work, help report results (including generation of tables or figures) or writing computer codes, should be stated. These should be listed in a cover letter to the editor and their use documented in appropriate sections of the manuscript, typically in the abstract, methods and acknowledgments sections^9,11,13,14^. Authors should be able to verify the accuracy of the entire manuscript and to assert that there is no plagiarism in their paper, including text and images produced by AI. Authors must personally ensure there is appropriate attribution of all quoted material, including full citations from original sources^13^.

Editors of biomedical journals should consider adopting the recent update of the ICJME (May 2023) recommendations for authorship and other aspects related to use of AI-assisted technologies for publication of biomedical papers^13^. WAME has recently recommended that editors and reviewers should specify to authors and to each other any use of chatbots in evaluation of manuscripts and generation of reviews and correspondence. WAME has emphasised that as the adverse consequences of misinformation include potential harm to people, editors of medical journals need access to appropriate digital tools to deal with the effects of chatbots on publishing, particularly content generated or altered by AI-assisted technologies^14^. An important principle is that while AI tools can aid scientific research and manuscript writing, it is crucial to recognise that they should function as a supplementary aid rather than a complete substitute for human creativity^6^.

In summary, ChatGPT and other AI-assisted technologies are here to stay and cannot be ignored. These AI tools are able to generate highly-realistic human-like text and perform a wide range of useful language tasks with a wide range of applications. It is key for us to learn how to manage its potential pitfalls and risks. AI tools have the potential to expedite innovation in health care and can aid in promoting equity and diversity in research by overcoming language barriers. The responsible use of AI language models and transparent reporting of how these tools are used in the creation of information and publication are vital to promote and protect the credibility and integrity of medical research, and trust in medical knowledge. When using these AI tools, authors must take responsibility for the output and originality of their work, as publishers expect all content to be generated by human authors unless there is a declaration to the contrary. Educating postgraduate and undergraduate students, researchers and authors on applications and best usage of AI-assisted technologies, together with the importance of critical thinking, integrity and strict adherence to ethical principles, are key steps that need to be undertaken.
